# A novel multiplex polymerase chain reaction assay for profile analyses of gene expression in peripheral blood

**DOI:** 10.1186/1471-2261-12-51

**Published:** 2012-07-10

**Authors:** Xingwang Jia, Haiyue Ju, Li Yang, Yaping Tian

**Affiliations:** 1 Department of Clinical Biochemistry, State Key Laboratory of Kidney Disease, Chinese PLA General Hospital, 100853, Beijing, China; 2 Department of Radiology, Chinese PLA General Hospital, 100853, Beijing, China

**Keywords:** Coronary artery disease, Gene expression profiling, Multiplex polymerase chain reaction, GeXP

## Abstract

**Background:**

Studies have demonstrated that inflammation has a key role in the pathogenesis of atherosclerosis due to the abnormal gene expressions of multiple cytokines. We established an accurate and precise method to observe gene expression in whole blood that might provide specific diagnostic information for coronary artery disease (CAD) and other related diseases.

**Methods:**

The fifteen selected CAD-related genes (IL1B, IL6, IL8, IFNG, MCP-1, VWF, MTHFR, SELL, TNFalpha, ubiquitin, MCSF, ICAM1, ID2, HMOX1 and LDLR) and two housekeeping genes (ACTB and GK) as internal references have been measured simultaneously with a newly developed multiplex polymerase chain reaction (multi-PCR) method. Moreover, the precision was evaluated, and a procedure for distinguishing patients from the normal population has been developed based upon analyses of peripheral blood. A total of 148 subjects were divided into group A (control group without plaques), group B (calcified plaques) and group C (non-calcified plaques, and combination group) according dual-source CT criteria. Gene expression in blood was analyzed by multi-PCR, and levels of glucose and lipids measured in 50 subjects to explore the relationship among them.

**Results:**

The precision results of the multi-PCR system revealed within-run and between-run CV values of 3.695–12.537% and 4.405–13.405%, respectively. The profiles of cytokine gene expression in peripheral blood were set: a positive correlation between glucose and MCSF, HMOX1 or TNFalpha were found. We also found that triglyceride levels were negatively correlated with SELL gene expression in 50 subjects. Compared with controls, gene expression levels of IL1B, IL6, IL8 and MCP-1 increased significantly in group C.

**Conclusions:**

A new multiple gene expression analysis system has been developed. The primary data suggested that gene expression was related to CAD. This system might be used for risk assessment of CVDs and other related diseases.

## Background

Coronary artery disease (CAD) is the main cause of death in adults in western countries [1]. During the past two decades, with changes in lifestyle and urbanization, the morbidity of CAD has increased gradually in China [2], and most subjects do not have symptoms before a cardiac event. Early recognition of the risk of atherosclerosis is important for the prevention of cardiac disease, as is early intervention in individuals at high risk of CAD. Nowadays, little information is available on early gene expression in subjects at high risk for CAD but who do not exhibit symptoms.

Evidence suggests that many acute coronary syndromes are caused by plaque disruption and thrombosis rather than stenosis severity [3]. The composition and configuration of atherosclerotic plaques are the main factors for plaque stability [4]. Conventional angiography, intravascular ultrasound (IVUS) and optical coherence tomography (OCT) are invasive or lack precision [5]. Multislice computed tomography (MSCT) is a new chapter in non-invasive assessment of atherosclerotic plaques, but its usage is restricted because of limited spatial resolution [6]. Hence, a reliable and non-invasive detection method is urgently needed in subjects who have risk factors for CAD. Peripheral blood is an accessible source compared with other tissues. Moreover, blood contains platelets, neutrophils and circulating leukocytes that are associated with processes in cardiovascular diseases (CVDs) [7]. Thus, gene expression profiling in peripheral blood could provide information on early risk factors for CVDs [8].

In recent years, studies have demonstrated that inflammation has a key role in the pathogenesis of atherosclerosis [9-11]. Multiple cytokines such as interleukin IL1B, IL6, IL8, IL10, IFNG, MCP-1, TNFalpha, MCSF, and ICAM1 are present at inflammation sites, each of which participate in the processes of atherosclerotic plaques [12-14]. Furthermore, TNFalpha, IL1B and IFNG promote the instability or disruption of atherosclerotic plaques [15]. Besides these inflammation-related cytokines, other gene expression panels such as HMOX1, VWF, ID2, MTHFR and SELL (which are also involved in the development and progression of atherosclerosis) are of great interest to researchers. However, almost all of such studies have focused on individual gene expression, which cannot provide the profile information of all inflammation-related cytokines [16-21].

Instead of the analyses of single genes described above, technologies such as cDNA microarrays can analyze thousand of transcripts in one chip [22]. However, it is expensive, has low sample throughput, and standardized procedures for optimization of the signal/noise ratio are lacking [23]. Beckman Coulter (Fullerton, CA, USA) developed the GenomeLab GeXP (Gene Expression Profiler) Genetic Analysis System that can be used to analyze up to 35 genes in a single reaction. Also, 192 samples can be evaluated simultaneously in a single analysis [24].

We carried out analytical validation of the GeXP system in blood samples. We were interested in establishing a platform for 15 CVD-related gene expression profiles and 2 housekeeping genes. The optimization process is described here.

## Methods

The study protocol was approved by the Medical Ethics Committee of the Chinese PLA General Hospital (Beijing, China). All subjects provided written informed consent to be included in the study.

### Patients and controls

According to the imaging characteristics of dual-source CT, subjects were divided into group A (control group without plaques), group B (calcified plaques) and group C (non-calcified plaques, and combination group). With respect to the classification criteria for the plaques, non-calcified plaques showed a density <150 Hounsfield units (HU) [25], whereas calcified plaques showed a density >300 HU [25,26]. The characteristics of the study subjects are shown in Additional file 1: Table 1.

### Sample collection and RNA extraction

Samples of peripheral blood were collected in ethylenediamine tetra-acetic acid (EDTA) vacutainer tubes (VACAETTE, Greiner, Austria). Ribonucleic Acid (RNA) was extracted within 6 h. One milliliter of whole blood was transferred to a new tube and 3 mL of Cell Lysis Solution (Promega, Fitchburg, WI, USA) was added to lyse red blood cells (RBC). The mixture was allowed to stand at room temperature for 10 min and was then centrifuged at 3,000 rpm for 3 min. Depositions were washed by isotonic NaCl and 1 mL of TRIzol reagent (Invitrogen, Carlsbad, CA, USA) added. After thorough mixing, 200 μL of chloroform was added. It was then centrifuged at 15,000 rpm for 10 min at room temperature. The upper aqueous phase was transferred to a 1.5-mL Eppendorf (Hamburg, Germany) tube and an equal volume of isopropanol added to precipitate RNA. The RNA pellet was resuspended in diethyl pyrocarbonate-treated water after it was washed with 75% ethanol and air-dried. Residual DNA was digested by Dnase (Promega). Finally, the total RNA concentration was quantified using a ultraviolet (UV) Spectrophotometer (Beckman Coulter) and RNA integrity checked by 1% agarose gel electrophoresis.

### Design of GeXPS primer

The multiplex primers were designed using Gexp express Profiler software (Beckman Coulter) (Additional file 2: Table 2). Each reverse primer contained a 19-nucleotide universal priming sequence at the 5′ end, and the 3′ end contained ≈20 nucleotides that were complementary to the target gene. Each forward primer consisted of a 18-nucleotide universal priming sequence at the 5′ end, and the 3′ end contained ≈20 nucleotides that corresponded to the target gene. Inside the Gexp PCR buffer were two pairs of primers labeled with D4 dye which were complementary to the 5′ end of the designed primers. All the PCR products were designed >3-bp apart, ranging from 137 bp to 287 bp, so that they could be distinguished by capillary electrophoresis analyses. In addition to the 15 genes of interest, each panel contained 2 housekeeping genes and Kanamycin RNA (Kan RNA) that served as an external control. Kan RNA can produce a peak at 325 bp after capillary electrophoresis. Besides that, two sets of primers for SELL were designed to examine the concordance of the product. The primers were synthesized and purified with PAGE method (TaKaRa, Shiga, Japan). All ordered primers were diluted to a final concentration of 500 nM (reverse primers) and 200 nM (forward primers) with nuclease-free water.

### Reverse transcription

RNA was diluted to 20–100 ng/μL. The reaction volume was 10 μL (containing 2 μL of 5 × RT buffers, 2.5 μL KanR RNA, 1 μL reverse primer, 0.5 μL Taq enzyme (Thermo Scientific, Waltham, MA, USA) and 1 μL RNA). In addition to the primer and Taq enzyme, other reagents were supported by a Genomelab GeXP Start Kit (Beckman Coulter). The RT reaction was done in a thermal cycler (Eppendorf) under the conditions: 1 min at 48°C, 60 min at 42°C, 5 min at 95°C, and hold at 4°C. Each experiment included a RT-Minus control and no-template control (NTC) to ensure each peak provided the expected result. The RT-Minus control was a no-enzyme reaction to ascertain if the RNA was contaminated with DNA. The NTC was a no-template control to confirm all that reaction reagents were in good condition.

### Polymerase chain reaction (PCR)

The reaction volume was 10 μL (2 μL of 5 × RT buffers, 2 μL 25 mM MgCl_2_, 0.35 μL of Thermo-Start DNA Polymerase, and 1.0 μL of forward primer plex) and 4.65 μL of cDNA samples from the RT plate. In addition to the primers, other reagents were supported by Thermo Scientific. The PCR reaction was done in a thermal cycler (Eppendorf) under the conditions: 10 min at 95°C, followed by 40 cycles of 30 s at 94°C, 30 s at 55°C, and 1 min at 68°C; hold at 4°C.

### Optimization of the concentration of the reverse primer

In the multiplex, there were extremely highly expressed genes and extremely poorly expressed genes. A dramatic reduction in the peak height of highly expressed genes could help to detect the very poorly expressed genes. A reference peak that had a median peak height of a multiplex was selected. All primers that had a peak height higher than the reference peak were pooled, and a twofold serial dilution made. An optimal dilution factor was achieved for primers when the signal strength of the high expressers was reduced close to that of a reference peak.

### Assessment of precision for the GeXP system

Two concentrations of total RNA were included in the experiment to assess precision. Ten samples were studied for assessment of intra-assay precision. Five days’ worth of tests were calculated for the inter-assay precision with starting RNA values of 33 ng and 66 ng.

### GeXP fragment and data analyses

The procedure for GeXP fragment and data analyses followed the manufacturer instructions. Briefly, PCR products from multiplex reactions were diluted 2:8 in water, and l μL added to the appropriate wells of a new 96-well sample microplate. A total of 0.5 μL DNA size standard 400 (GenomeLab GeXP Start Kit; Beckman Coulter) was added to 38.5 μL of sample loading solution with thorough mixing. The mix solution was assembled and added to the 96-well sample microplate. The PCR product was separated based on fragment size by capillary gel electrophoresis. The strength of the dye signal was measured after normalization to KANr RNA using the area under the curve (AUC). Normalized data were exported to Microsoft Excel for further analyses.

### Serum measurements

A commercially available enzymatic kit (Roche Diagnostics, Basel Switzerland) was used to measure the serum concentration of glucose, total cholesterol (TC), triglyceride (TG), high-density lipoprotein cholesterol (HDL-C), and low-density lipoprotein cholesterol (LDL-C). The inter-assay of variation (CVs) were <1.9% for glucose, <0.8% for TC, <2.4% for TG, <1.85% for HDL-C, and <1.20% for LDL-C.

### Statistical analyses

Differences in gene expression between groups were compared using the Mann–Whitney non-parametric test. Spearman’s correlations were used in study of correlation analyses. *P* < 0.05 was considered significant.

## Results

### Single PCR

**Figure 1:**
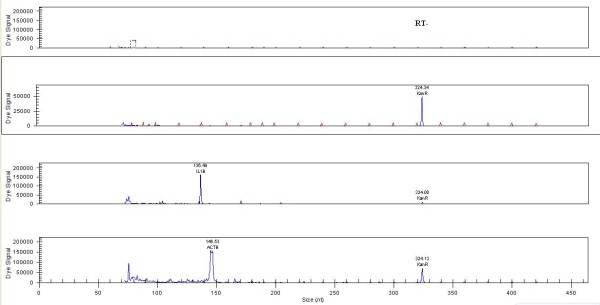
**Single RT-PCR capillary gel electrophoresis of the selected genes.** Single RT-PCR capillary gel electrophoresis results of RT-PCR, NTC, IL1B and ACTB. Other results can be found in Additional file 3, 4, 5 and 6.

Each pair of primers was validated by a single PCR (Figure 1 Additional file 3, 4, 5 and 6). All the products were approximately equal to the designed size, demonstrating that all the genes were amplified successfully. Kan RNA with 325-bp peak was detected in all the single PCRs. NTC and RT-Minus control results showed that all reaction reagents were in good condition and that the starting RNA was free of DNA.

### Optimized results for reverse primers

**Figure 2:**
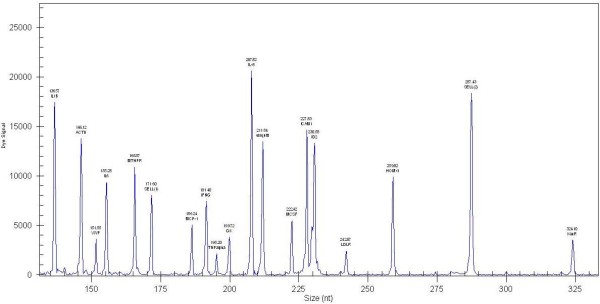
**Multiplex primer RT-PCR capillary gel electrophoresis results before and after optimization.** Multiplex primer RT-PCR capillary gel electrophoresis results after optimization. Multiplex primer RT-PCR capillary gel electrophoresis results before optimization can be found in Additional file 7.

Our results showed that the reverse primer of four genes should be diluted and that four genes should be concentrated. The appropriate dilutions of the SELL (1), IL1B, ubiquitin and ACTB genes were 1:2, 1:4, 1:4 and 1:16, respectively. The appropriate concentrations of MTHFR, MCSF and LDLR were twofold and that of TNFalpha was threefold. Capillary gel electrophoresis results are shown in Figure 2.

### Assessment of precision for the GeXP system

The precision experiment showed that the within-run and between-run CV values were 3.695–12.537% and 4.405–13.405%, respectively (Additional file 8: Table 3).

### Comparison between different products amplified by different primers for the same gene

**Figure 3:**
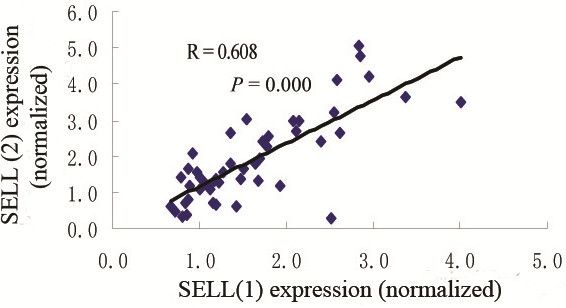
Comparison between different products amplified by different primers for SELL.

To validate the stability and concordance of the GeXP system, two sets of primers for SELL were designed. The correlations between the two products were compared (Figure 3). The correlation efficient between the two products was 0.780 (*P* = 0.000).

### Correlation analyses between gene expression in peripheral blood and biochemical items

**Figure 4:**
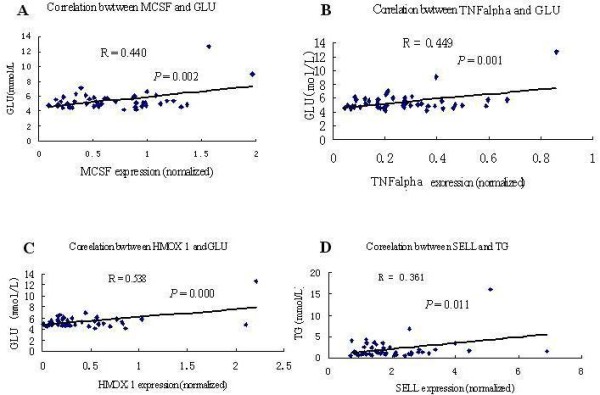
**Correlations between gene expression in peripheral blood and biochemical items.** The correlation coefficients between MCSF and glucose (**A**), TNFalpha and glucose (**B**), HMOX1 and glucose (**C**) were 0.440, 0.449 and 0.538, respectively (P < 0.01). The correlation coefficient between SELL and triglyceride was 0.361 (P < 0.05).

We carried out the optimized assay to test blood samples from 50 volunteers. We analyzed the correlation between gene expression and biochemical items. Three gene expression levels correlated with serum glucose: TNF-α, MCSF and HMOX1. Moreover, SELL correlated with TG (Figure 4)

### Diagnosis value of the selected gene

**Figure 5:**
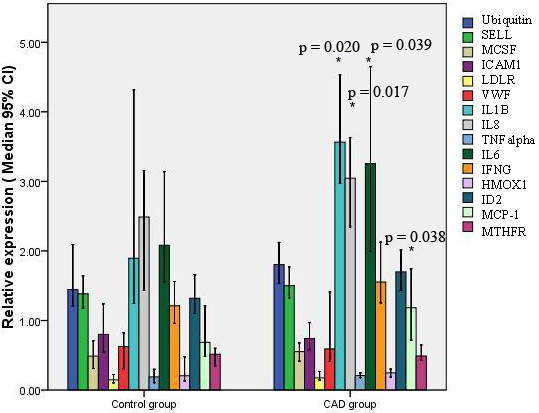
**Relative expression of 15 CAD-related genes in the CAD group and control group.** Data were normalized to ACTB and GK. Compared with control group, *P < 0.05.

We detected a 15-gene expression profile in group C and the control group with the method described above. The expressions of IL1B, IL6, IL8, and MCP1 were significantly different between group C and the control group (Figure 5). The combination of the four markers helped to discriminate patients from controls (Additional file 9: Table 4).

### Validation of the findings

**Figure 6:**
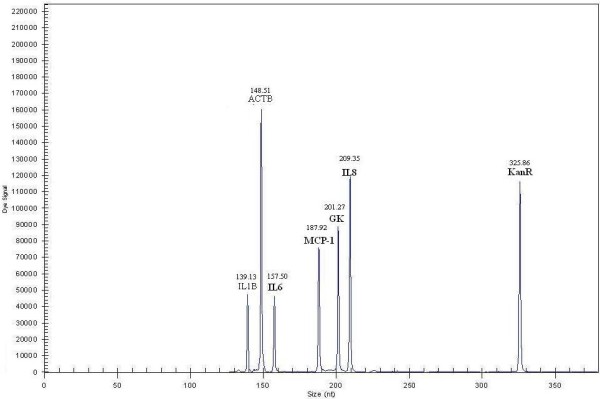
Multiplex primer RT-PCR capillary gel electrophoresis results of 4 CAD-related genes.

Another independent set of 15 patients and 15 controls were used to validate our previous findings. The results of multiplex primer RT-PCR capillary gel electrophoresis of four CAD-related genes are shown in Figure 6. The p values of IL1B, IL6, IL8 and MCP-1 between these two groups were 0.023, 0.014, 0.021, and 0.048, respectively. These results further confirmed that IL1B, IL6, IL8 and MCP-1 were the cytokines most closely related with CAD.

### Comparison of the positive and negative predictive values of single genes and four markers

We calculated the positive and negative predictive values of single genes and four markers .The results were also validated in independent sample (Additional file 10: Table 5).

### Procedure for distinguishing between the CAD state and normal state using the GeXP system

**Figure 7:**
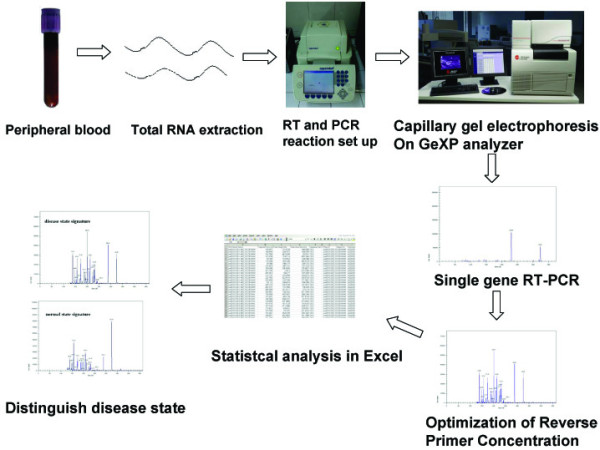
**Procedure for distinguishing a CAD state or normal state based on peripheral blood using the GeXP system.** The procedure involved the following steps: extraction of total RNA; RT and PCR reaction setup; single gene RT-PCR; multiplex primer RT-PCR and optimization; and statistical analyses.

Therefore, by using the GeXP system, we could distinguish a CAD state and a normal state (Figure 7). The methodology involved the following steps: extraction of total RNA; RT and PCR reaction setup; single gene RT-PCR; multiplex primer RT-PCR and optimization; and statistical analyses. Significant gene panels can be observed for further study.

## Discussion

How to use low amounts of RNA to amplify multiplex genes in a single reaction is a controversial subject. Real-time PCR can be used to analyze the expression of one or a few genes, which is not suitable for multi-PCR detection. Gene chip technology has high throughput, but it is not suitable for batch-sample detection in clinical laboratories due to its high cost and low reproducibility. In recent years, GeXP technology has been used in research in viruses, animals and humans [27-31]. The multi-PCR assay using GeXP technology has been validated by real-time PCR [32].

We therefore developed a rapid multi-PCR assay for analyses of gene expression profiling in peripheral blood. According to the literature, ACTB and GK are chosen as reference genes [33-35]. The entire product was equal to the designed size, which demonstrated that all the target genes were amplified successfully. This finding showed that this assay could be used for small samples, had good reproducibility, and was easy to operate. The results indicated that this multi-PCR assay might provide profile analyses of gene expression in peripheral blood for the risk assessment of CVDs and other inflammation-related diseases. However, the multiplex must be optimized individually by attenuation. Furthermore, a lack of precision was present in other processes, such as RNA extraction, RT, and the PCR reaction. A standard procedure is needed to reduce the chance of experimental errors.

Research has shown that high levels of plasma lipids may promote atherosclerosis [36] and that levels of pro-inflammatory mediators are increased in the early phases of diabetes [37]. The present study also showed that glucose was positively correlated with the expression of MCSF, HMOX-1 or TNF-α, and that TG was negatively correlated with SELL gene expression. The relationship between gene expression and biochemical items merits further study.

Previous studies indicated that all 15 selected CAD-related genes might have an important role in the pathogenesis of atherosclerosis. However, there were few reports about the relationship between these genes. Therefore, we developed a new multiplex PCR assay to measure them together in one reaction system. We then compared which genes had the more important roles in relation to CAD. The results showed that only 4 from 15 analyzed genes were significantly altered when comparing group C with group A. The panel of 4 genes confirmed these results in an independent set of tests.

The Diamond–Forrester method uses age, sex and symptoms to provide a quantitative assessment of the probability of coronary disease [38]. CAD is distinguished by narrowing of the luminal diameter of vessels. However, many acute coronary syndromes are caused by plaque disruption and thrombosis rather than stenosis severity. Inflammatory mediators such as IL1B, IL8 and MCP-1 are produced by monocytes and macrophages and they are responsible to modifying LDL and/or killing bacterial products, whereas IL6 is made within plaques by macrophages and endothelial cells [12]. Overexpression of these four genes would be involved in the pathological processes of the cardiovascular system, and might accelerate the formation of arterial plaques. Traditional clinical factors are CAD risk factors, and have not been used to distinguish the characteristics and compositions of plaques. Hence, we set up a non-invasive detection method using the expression patterns of four genes in peripheral blood.

The AUC of the four-gene model was modest at best, with poor sensitivity and high specificity. Furthermore, the validation results showed the positive and negative predictive values of the four markers are 71.4% and 68.7% respectively. These results may be meaningful for the assessment of CAD in laboratory-based studies. Subjects with symptomatic CAD agree to undergo conventional angiography, but most individuals without symptomatic CAD accept conventional angiography reluctantly. Our model can be used in the assessment of subjects with asymptomatic CAD. If someone is initially diagnosed as having CAD, then imaging is necessary as the next step for the four-gene model. This technology might hold promise in the identification of patients with non-calcified plaques, but it has limitations (e.g., insufficient sensitivity).

## Conclusions

In summary, a new multiple gene expression analysis system has been developed. The primary data indicated that this system might provide some information for identifying patients with non-calcified plaques. We welcome further evaluation of this method in larger study cohorts.

## Abbreviations

IL1B: Interleukin-1-beta; IL6: Interleukin-6; IL8: Interleukin-8; IFNG: Interferon-gamma; MCP-1: Monocyte chemoattractant protein-1; TNFalpha: Tumor necrosis factor-alpha; VWF: Von Wi1lebrand factor; MTHFR: Methylene tetrahydrofolate reductase; ICAM1: Intercellular adhesion molecule-1; HMOX1: Heme oxygenase-1; LDLR: Low-density lipoprotein receptor; GK: Sapiens glycerol kinase; ID2: Inhibitors of DNA Binding 2; SELL: L-selectin; ACTB: Actin, beta; GeXP: Gene Expression Profiler; CAD: Coronary artery disease; AUC: Area under the curve.

## Competing interests

The authors declare that they have no competing interests.

## Authors’ contributions

XJ designed the study, undertook the statistical analysis, data collection and literature search, and drafted the manuscript. HJ analyzed the imaging characteristics of the subjects using dual-source CT. LY participated in the study design. YT participated in the study design, fund collection and coordination, and helped to draft the manuscript. All authors read and approved the final manuscript.

## Authors’ information

Xingwang Jia has a doctorate in the diagnostic use of clinical biochemistry, and is a Senior Technician-in-Charge in the Department of Biochemistry of Chinese PLA General Hospital.

Haiyue Ju has a doctorate in the field of radiological diagnosis, and is an attending physician in the Department of Radiology of Chinese PLA General Hospital.

Li Yang is the Director in the Department of Radiology of Chinese PLA General Hospital, and is a leader in the field of radiological diagnosis in China.

Yaping Tian is the Director in the Department of Clinical Biochemistry of Chinese PLA General Hospital, and is a leader in the field of clinical laboratory examinations in China.

## Pre-publication history

The pre-publication history for this paper can be accessed here:

http://www.biomedcentral.com/1471-2261/12/51/prepub

## Supplementary Material

Additional file 1 **Table 1.** Characteristics of the study subjects. Click here for file

Additional file 2 **Table 2.** Genes and GeXP designed primers in the multiplex RT-PCR. Click here for file

Additional file 3 Single RT-PCR capillary gel electrophoresis results of IFNG, ubiquitin, GK and IL8. Click here for file

Additional file 4 Single RT-PCR capillary gel electrophoresis results of TNFalpha, ICAM1, ID2 and LDLR. Click here for file

Additional file 5 Single RT-PCR capillary gel electrophoresis results of MCSF, HMOX1, MCP-1 and SELL (2). Click here for file

Additional file 6 Single RT-PCR capillary gel electrophoresis results of IL6, VWF, SELL (1) and MTHFR. Click here for file

Additional file 7 Multiplex primer RT-PCR capillary gel electrophoresis results before optimization Click here for file

Additional file 8 **Table 3.** Precision assessment for the GeXP analyzer. Click here for file

Additional file 9 **Table 4.** Comparison of the diagnostic effects of single genes and four markers. Click here for file

Additional file 10 **Table 5.** Comparison of the positive and negative predictive values of single genes and four markers. Click here for file
