# Supplementary material for: A novel multiplex polymerase chain reaction assay for profile analyses of gene expression in peripheral blood
Source: BMC Cardiovasc Disord. 2012 Jul 10;12:51. doi: 10.1186/1471-2261-12-51 (PMC3445828; doi:10.1186/1471-2261-12-51)
Supplement: Additional file 1 — Table 1. Characteristics of the study subjects. [file 1471-2261-12-51-S1.doc]

**Table1. Characteristics of the study subjects**

| Group | A | B | C | |
| --- | --- | --- | --- | --- |
|  | Control group | Calcified plaque | Non-calcified plaque | Combination group |
| N | 44 | 15 | 45 | 44 |
| M/F | 32/12* | 8/7 | 32/13 | 38/6 |
| Age (years) | 50.1±10.7 * | 55.4±8.5 | 48.3±11.2 | 55.3 ±10.3 |
| Smoking | 15* | 4 | 15 | 24 |
| Diabetes mellitus | 8* | 3 | 10 | 10 |
| Hypertension | 2* | 1 | 3 | 2 |
| Hyperlipidemia | 15* | 11 | 16 | 15 |
| ACEI treatment | 2* | 1 | 3 | 2 |
| Statin treatment | 2* | 1 | 2 | 3 |

ACEI, angiotensin-converting enzyme inhibitor
Age is expressed as mean ± SD.
*P>0.05
