# Supplementary material for: A novel multiplex polymerase chain reaction assay for profile analyses of gene expression in peripheral blood
Source: BMC Cardiovasc Disord. 2012 Jul 10;12:51. doi: 10.1186/1471-2261-12-51 (PMC3445828; doi:10.1186/1471-2261-12-51)
Supplement: Additional file 2 — Table 2. Genes and GeXP designed primers in the multiplex RT-PCR. [file 1471-2261-12-51-S2.doc]

Table 2. Genes and GeXP designed primers in the multiplex RT-PCR

| **Gene symbol** | **Ref Sequence** | **Reverse primer (5′→3′)** | **Forward primer (5′→3′)** | **Size of product** |
| --- | --- | --- | --- | --- |
| [IL1B](http://www.genenames.org/data/hgnc_data.php?hgnc_id=5992) | NM_000576 | GTACGACTCACTATAGGGAGTGGTCGGAGATTCGTAGCT | AGGTGACACTATAGAATAGTTCTTTGAAGCTGATGGCC | 137 |
| [ACTB](http://www.genenames.org/data/hgnc_data.php?hgnc_id=132)^1^ | NM_001101 | GTACGACTCACTATAGGGAGTCAGGCAGCTCGTAGCTCT | AGGTGACACTATAGAATATCGTGCGTGACATTAAGGAG | 147 |
| VWF | NM_000552 | GTACGACTCACTATAGGGACAAGATACACGGAGAGGCTC | AGGTGACACTATAGAATATACAGCTTTGCGGGATACTG | 152 |
| IL6 | NM_000600.3 | GTACGACTCACTATAGGGAGCCATCTTTGGAAGGTTCAG | AGGTGACACTATAGAATAAATTCGGTACATCCTCGACG | 157 |
| MTHFR | NM_005957 | GTACGACTCACTATAGGGATCTTCTCCCGGAGTCTCTCA | AGGTGACACTATAGAATAAGAGGAAACAGCAGCCTCAA | 167 |
| MCP-1 | NM_002982.3 | GTACGACTCACTATAGGGAGGGTTGTGGAGTGAGTGTTC | AGGTGACACTATAGAATAAGCAAGTGTCCCAAAGAAGC | 187 |
| IFNG | NM_000619 | GTACGACTCACTATAGGGAGACAGTTCAGCCATCACTTGG | AGGTGACACTATAGAATAGTGGAGACCATCAAGGAAGAC | 192 |
| IL8 | NM_000584 | GTACGACTCACTATAGGGAAGCAGACTAGGGTTGCCAGA | AGGTGACACTATAGAATAAAGGGCCAAGAGAATATCCG | 207 |
| TNFalpha | NM_000594 | GTACGACTCACTATAGGGACAAAGTCGAGATAGTCGGGC | AGGTGACACTATAGAATAAACCTCCTCTCTGCCATCAA | 197 |
| Ubiquitin | M26880 | GTACGACTCACTATAGGGAAACCTTAGGTGGTTTGAAGGG | AGGTGACACTATAGAATAACTTAGTGATTTGGCCCGTG | 212 |
| MCSF | NM_172212.2 | GTACGACTCACTATAGGGATCTCTGAAGCGCATGGTGT | AGGTGACACTATAGAATAAGCCACATGATTGGGAGTG | 222 |
| ID2 | NM_002166 | GTACGACTCACTATAGGGAGAGAACACCCTGGGAAGATG | AGGTGACACTATAGAATAGTGGCTGAATAAGCGGTGTT | 231 |
| GK^1^ | NM_203391 | GTACGACTCACTATAGGGATTGATGTTAAGAAGCAGGATTAGG | AGGTGACACTATAGAATAAGAAATTGCTATCTGGGATGACA | 201 |
| ICAM1 | NM_000201 | GTACGACTCACTATAGGGATCAAGGGTTGGGGTCAGTAG | AGGTGACACTATAGAATAAGACATAGCCCCACCATGAG | 227 |
| LDLR | Nm_000527 | GTACGACTCACTATAGGGAGTTGGCACTGAAAATGGCTT | AGGTGACACTATAGAATATGGCATCACCCTAGATCTCC | 240 |
| HMOX1 | NM_002133 | GTACGACTCACTATAGGGAACTGTCGCCACCAGAAAGCT | AGGTGACACTATAGAATAGTTCCTGCTCAACATCCAGC | 262 |
| SELL (1) | NM_000655 | GTACGACTCACTATAGGGACTTTCACCAAGGGCGATTTA | AGGTGACACTATAGAATACTTCATTCCAGTGGCAGTCA | 172 |
| SELL (2) | NM_000655 | GTACGACTCACTATAGGGATGCAGAGGTAAAGCTGAAGCTGGC | AGGTGACACTATAGAATAGGCCCCAGAGCTGGGTACCAT | 287 |

Underlined oligonucleotides are universal sequences.

^1^ Gene used for normalization
