# Supplementary material for: A novel multiplex polymerase chain reaction assay for profile analyses of gene expression in peripheral blood
Source: BMC Cardiovasc Disord. 2012 Jul 10;12:51. doi: 10.1186/1471-2261-12-51 (PMC3445828; doi:10.1186/1471-2261-12-51)
Supplement: Additional file 3 — Single RT-PCR capillary gel electrophoresis results of IFNG, ubiquitin, GK and IL8. [file 1471-2261-12-51-S3.doc]

Table 3. Precision assessment for the GeXP analyzer

| Precision experiment (CV%) | n | 33 ng | 66 ng |
| --- | --- | --- | --- |
| Within-run | 10 | 3.695(1.372 –7.200) | 12.537(2.616–15.303) |
| Between–run | 5 | 4.405(1.392 –9.945) | 13.405(2.749–14.726) |
