# Supplementary material for: A novel multiplex polymerase chain reaction assay for profile analyses of gene expression in peripheral blood
Source: BMC Cardiovasc Disord. 2012 Jul 10;12:51. doi: 10.1186/1471-2261-12-51 (PMC3445828; doi:10.1186/1471-2261-12-51)
Supplement: Additional file 4 — Single RT-PCR capillary gel electrophoresis results of TNFalpha, ICAM1, ID2 and LDLR. [file 1471-2261-12-51-S4.doc]

Table 4. Comparison of the diagnostic effects of single genes and four markers

| Gene | Threshold | Sensitivity (%) | Specificity (%) | AUC (95% CI) |
| --- | --- | --- | --- | --- |
| [IL1B](http://www.genenames.org/data/hgnc_data.php?hgnc_id=5992) | 1.659 | 85.5 | 50.0 | 0.639 (0.539 - 0.730) |
| IL6 | 3.958 | 43.5 | 91.7 | 0.623 (0.523 - 0.716) |
| IL8 | 3.330 | 47.8 | 80.6 | 0.642 (0.542 - 0.733) |
| MCP-1 | 1.412 | 49.3 | 80.6 | 0.624 (0.524 - 0.717) |
| [IL1B](http://www.genenames.org/data/hgnc_data.php?hgnc_id=5992)+IL6+ IL8+MCP-1 | 0.485 | 50.7 | 77.8 | 0.669 (0.570 - 0.757) |
| Validation([IL1B](http://www.genenames.org/data/hgnc_data.php?hgnc_id=5992)+IL6  +IL8+MCP-1) | 0.403 | 66.6 | 73.3 | 0.830(0. 650 - 0.942) |

AUC, area under the curve
