# Supplementary material for: A novel multiplex polymerase chain reaction assay for profile analyses of gene expression in peripheral blood
Source: BMC Cardiovasc Disord. 2012 Jul 10;12:51. doi: 10.1186/1471-2261-12-51 (PMC3445828; doi:10.1186/1471-2261-12-51)
Supplement: Additional file 5 — Single RT-PCR capillary gel electrophoresis results of MCSF, HMOX1, MCP-1 and SELL (2). [file 1471-2261-12-51-S5.doc]

Table 5. Comparison of the positive and negative predictive values of single genes and four markers

| Gene | positive predictive values | negative predictive values |
| --- | --- | --- |
| [IL1B](http://www.genenames.org/data/hgnc_data.php?hgnc_id=5992) | 76.6 | 64.3 |
| IL6 | 90.9 | 45.8 |
| IL8 | 82.5 | 44.6 |
| MCP-1 | 82.9 | 45.3 |
| [IL1B](http://www.genenames.org/data/hgnc_data.php?hgnc_id=5992)+IL6+IL8+MCP-1 | 81.4 | 45.2 |
| Validation ([IL1B](http://www.genenames.org/data/hgnc_data.php?hgnc_id=5992)+IL6+IL8+MCP-1) | 71.4 | 68.7 |
