# Supplementary figures and images for: A novel multiplex polymerase chain reaction assay for profile analyses of gene expression in peripheral blood
Source: BMC Cardiovasc Disord. 2012 Jul 10;12:51. doi: 10.1186/1471-2261-12-51 (PMC3445828; doi:10.1186/1471-2261-12-51)

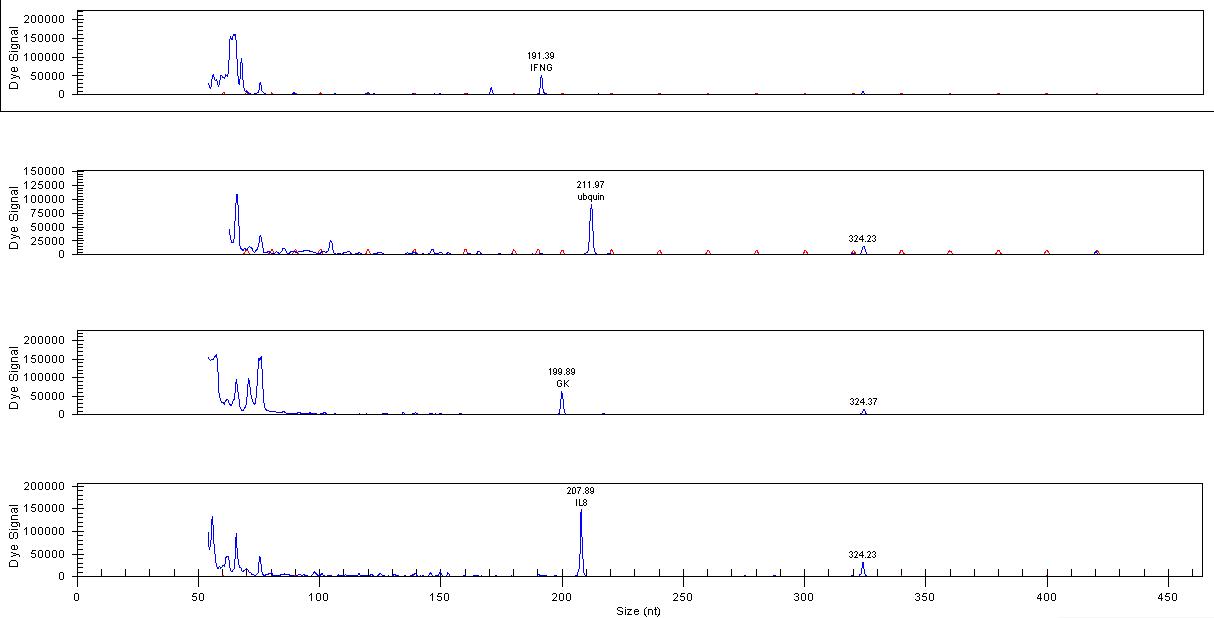

Supplement: Additional file 6 — Single RT-PCR capillary gel electrophoresis results of IL6, VWF, SELL (1) and MTHFR. [file 1471-2261-12-51-S6.jpeg]

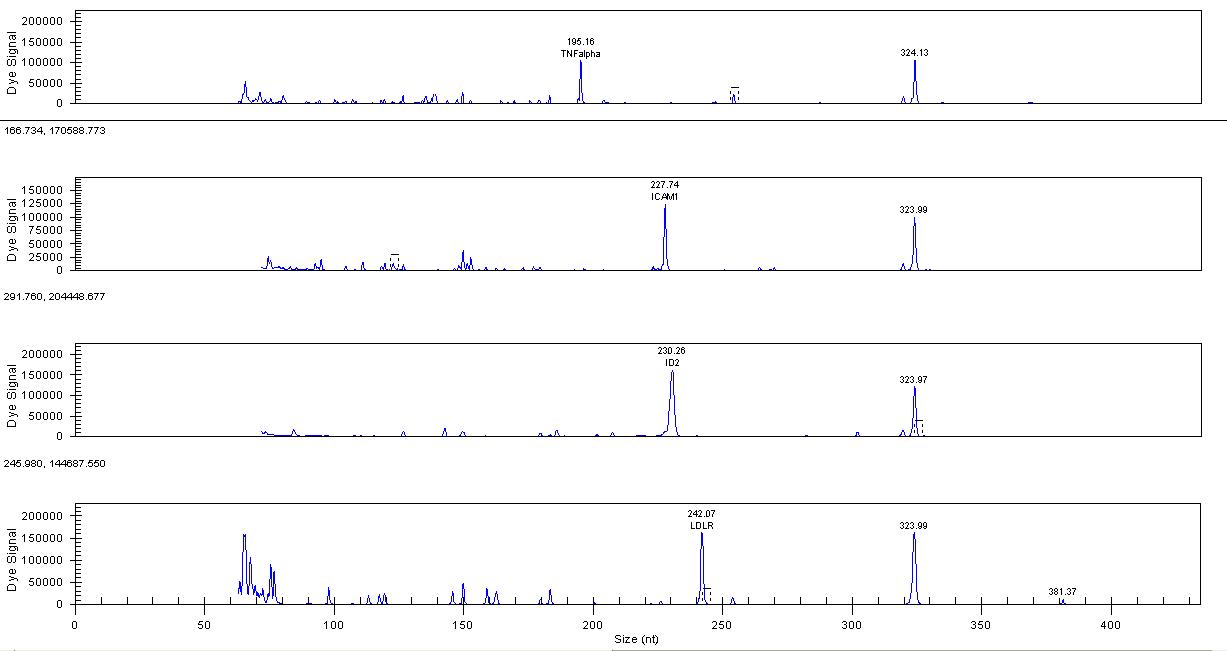

Supplement: Additional file 7 — Multiplex primer RT-PCR capillary gel electrophoresis results before optimization [file 1471-2261-12-51-S7.jpeg]

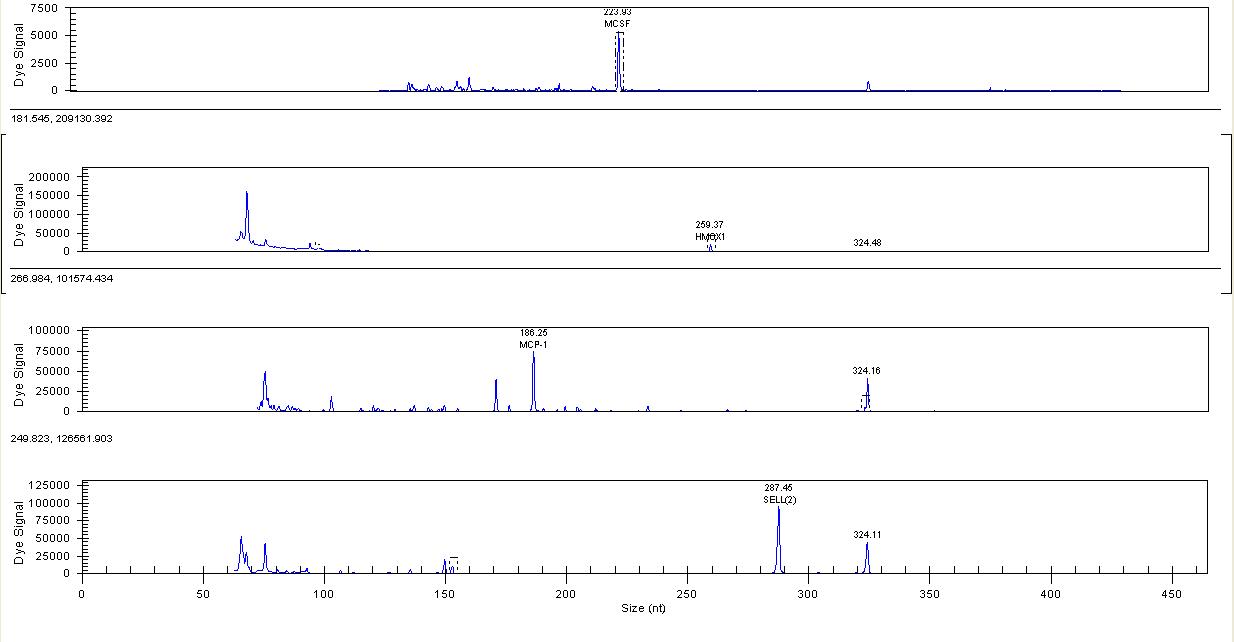

Supplement: Additional file 8 — Table 3. Precision assessment for the GeXP analyzer. [file 1471-2261-12-51-S8.jpeg]

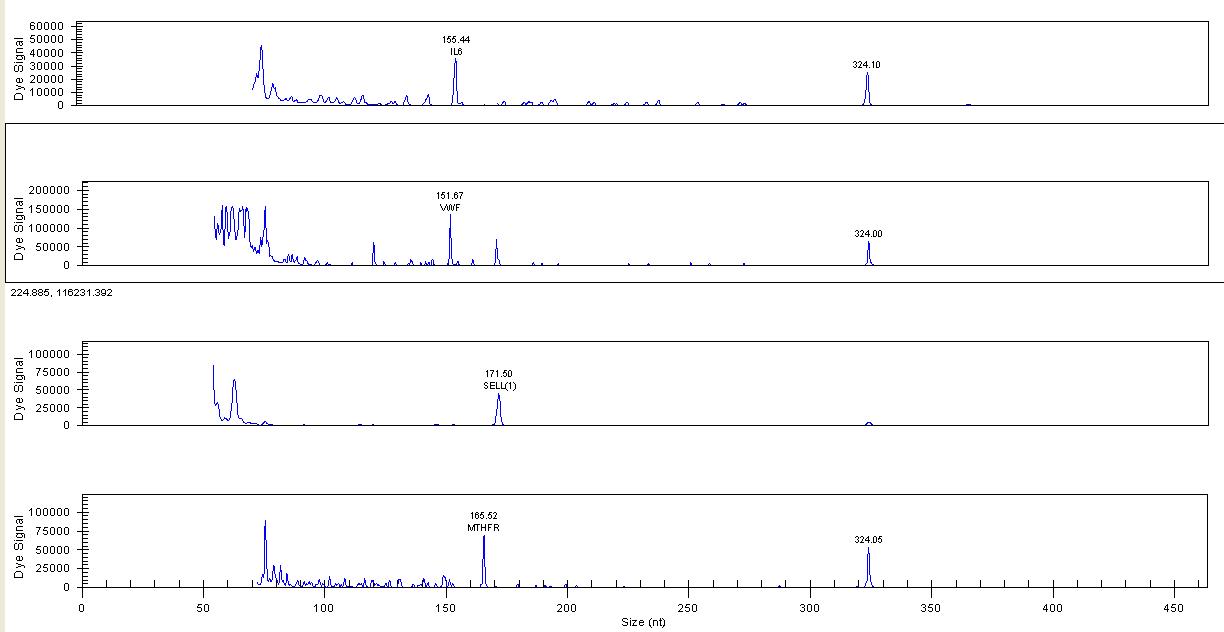

Supplement: Additional file 9 — Table 4. Comparison of the diagnostic effects of single genes and four markers. [file 1471-2261-12-51-S9.jpeg]

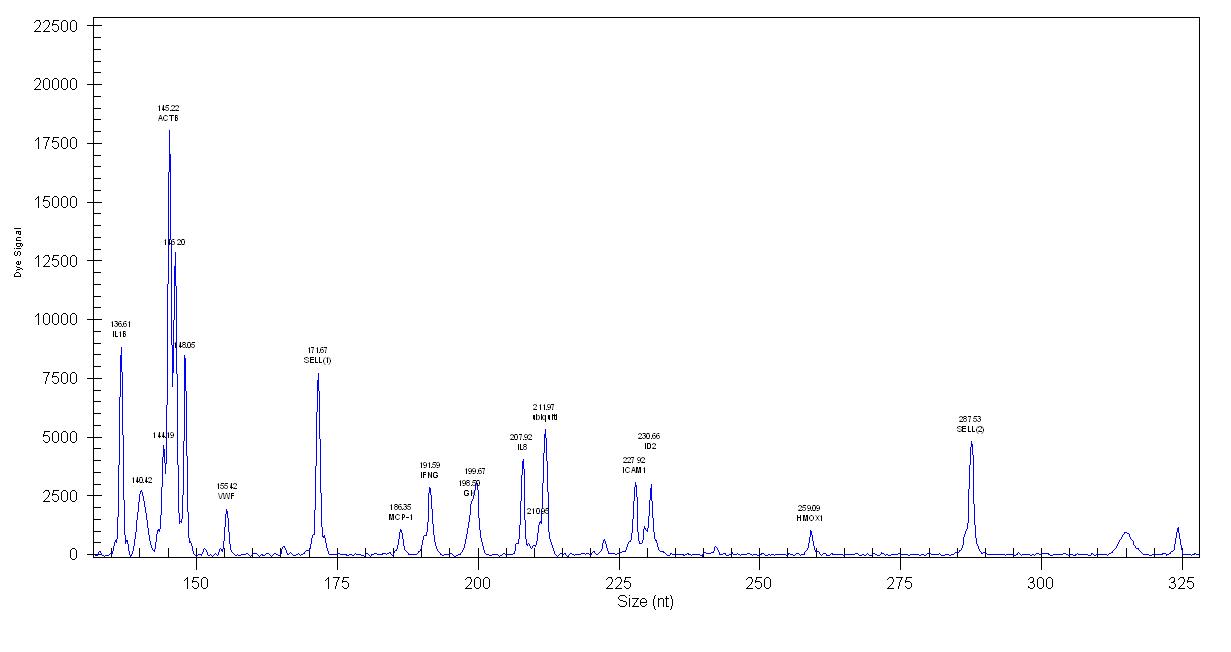

Supplement: Additional file 10 — Table 5. Comparison of the positive and negative predictive values of single genes and four markers. [file 1471-2261-12-51-S10.jpeg]
